# Activation of liver X receptor delayed the retinal degeneration of rd1 mice through modulation of the immunological function of glia

**DOI:** 10.18632/oncotarget.16643

**Published:** 2017-03-29

**Authors:** Xiao He, Dayu Sun, Siyu Chen, Haiwei Xu

**Affiliations:** ^1^ Southwest Hospital/Southwest Eye Hospital, Third Military Medical University, Chongqing, 400038, China; ^2^ Key Lab of Visual Damage and Regeneration & Restoration of Chongqing, Chongqing, 400038, China

**Keywords:** retinal degeneration, liver X receptors, JAK-STAT pathway, microglia, apoptosis

## Abstract

Retinal degeneration (RD), including retinitis pigmentosa (RP), is an inherited eye disease characterized by progressive degeneration of photoreceptors. Recently, immune cells, including microglia, Müller cells and astrocytes, in degenerative retina are demonstrated to play key roles in the development of RD and can be used as potential therapeutic targets. Liver X receptors (LXRs) are important immuno-inflammatory response transcription factors that have been reported to be a new potential therapeutic drug target for neurodegenerative diseases. However, the potential therapeutic utility of LXRs for RP has not been evaluated. In the present study, Pde6β (rd1) mice received intraperitoneal injections of T0901317 (T0, 50 mg/kg/d) or vehicle (2% DMSO) for 7 days with age-matched C57/BL6 mice as controls. The effect of T0 was examined by quantitating photoreceptor apoptosis, microglial density and the expression of inflammatory mediators; the underlying mechanisms were then explored with a microarray assay. T0 markedly delayed apoptosis of the photoreceptors, partially through suppressing the activation of microglia and the gliosis of Müller cells, and decreased the expression levels of IL-6, iNOS, COX-2 and ENG in rd1 mice; as a result, the visual function of T0-treated rd1 mice measured with electroretinograms (ERG) was preserved for a longer time than that of vehicle-treated rd1 mice. The microarray assay showed that the Janus kinase/Signal Transducer and Activator of Transcription (JAK-STAT) signaling pathway was significantly affected in the retina of rd1 mice with T0 treatment. Our data suggested that T0 modulated the immunologic function of glia cells in the degenerative retina through the JAK3/STAT pathway and delayed the apoptosis of photoreceptors.

## INTRODUCTION

Inherited retinal degeneration (RD), including retinitis pigmentosa (RP), is characterized by selective retinal photoreceptor cell degeneration and apoptosis [1] [2]. RP is one of the prevalent blinding diseases in developed countries; currently, there is no comprehensive treatment to cure RP [3, 4]. More than 100 genes have been reported to cause RP [3]; therefore, it is necessary to explore the broadly shared mechanisms of RP rather than the individual mutations.

Recently, inflammation [5–9] is found to play a fundamental role in photoreceptor degeneration in the RP animal model and RP patients. Microglia are the only resident innate immune cells in the retina, and activation of microglia in the retina is considered a contributing factor in early stages of RP [8], demonstrating that microglia can be used as a potential therapeutic target of RD [10]. Suppressing the activation of microglia has been proven to reduce the apoptosis of photoreceptors in rd10 mice [11]. During the development of retinal degeneration, the Müller cell and astrocyte processes usually become thickened and enlarged as a response to neuro-degeneration, usually causing reactive gliosis [12, 13] and preventing the regeneration of the retina.

Liver X receptors (LXRs) are a nuclear receptor superfamily and a type of ligand-activated transcription factor that include two isoforms, LXRα and LXRβ [14, 15]. In addition to being involved in the regulation of lipid metabolism, LXRs have been reported to play important roles in the modulation of inflammation [14]. Our previous results demonstrate that activation of LXRs usually downregulate pro-inflammatory signals in the central nervous system (CNS) [14, 16]. Most recently, agonists of LXRs T0901317 (T0) and GW3965 are reported to induce the expression of genes coding for activators of photoreceptor guanylate cyclase and the subunit of the cGMP-specific phosphodiesterase complex (Pde6h) in the neural retina of zebrafish [17], suggesting that LXRs are involved in the development of the retina and may be an important component of the retinal metabolism necessary for the maintenance of visual function [18, 19]. It has been reported that activation of LXRs with the synthetic agonist T0 could protect inner retinal damage induced by N-methyl-D-aspartate [20] or alleviate ocular inflammation in experimental autoimmune uveitis [21].

The Pde6β (rd1) mouse, the best-characterized classical mouse model of human RP, carries a mutation in cyclic guanidine monophosphate (cGMP)–phosphodiesterase 6β (Pde6β) in the rod photoreceptors. The dysfunction of Pde6β results in the accumulation of cGMP and causes the degeneration and apoptosis of the rod photoreceptors [14]. It is not clear whether T0 could delay the degeneration process of photoreceptors in the rd1 mouse model.

In the present study, we observed the effect of T0 on the activation of microglia and Müller cells in the degenerative retina and its influence on the expression of cytokines including IL-6, iNOS, COX-2 and ENG, as well as the apoptosis of photoreceptors. The function of the retina of rd1 mice was tested with electrophysiology (ERG). A microarray analysis was used to test the gene expression profiles of the retina with T0 treatment of rd1 mice. We aimed to explore the immunological function of LXRs activation and its neuroprotective effects on photoreceptors during the development of RD as well as its underlying mechanisms.

## RESULTS

### T0 reduces photoreceptor apoptosis and delays retinal degeneration in the rd1 mice

**Figure 1:**
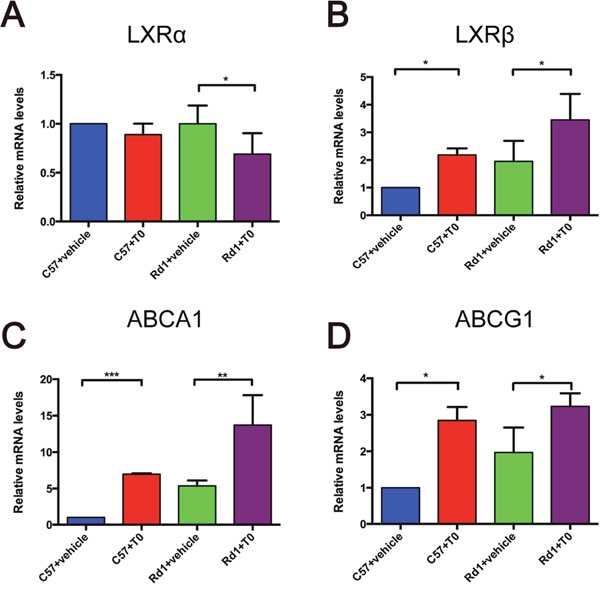
Effect of T0901317 on the expression of LXRα and LXRβ and their target genes ABCA1 and ABCG1 in mouse retina mRNA levels were measured at P14. **(A, B)** LXRα and LXRβ expression levels in C57 and rd1 mice. The LXRα mRNA expression level is decreased by T0 treatment in rd1 mice, whereas there is no significant difference between T0 treated and non-treated C57 mice. LXRβ mRNA expression levels is increased by T0 treatment in both groups of mice. **(C, D)** ABCA1 and ABCG1 expression levels in C57 and rd1 mice. Both genes levels are increased by T0 treatment compared with vehicle treatment. n=4 in each group. ***P<0.001, **P<0.01, *P<0.05.

As previously reported [21, 22], both LXRα and LXRβ are expressed in the retina of C57 mice at postnatal day (P) 14. The mRNA level of LXRα was lower in the retina of rd1 mice compared to C57 mice at P14 [22]. In present study, after intraperitoneal (i.p.) injection of T0 for 7 successive days, the LXRα mRNA level in the retina of rd1 mice is reduced compared to their controls (Figure 1A), while the LXRβ mRNA level in the retina of both C57 and rd1 mice is increased in comparison with those injected with vehicle (Figure 1B). The expression of the target genes of LXRs ABCA1 and ABCG1 in C57 and rd1 mice is upregulated by T0 treatment compared with vehicle treatment (Figure 1C, 1D).

**Figure 2:**
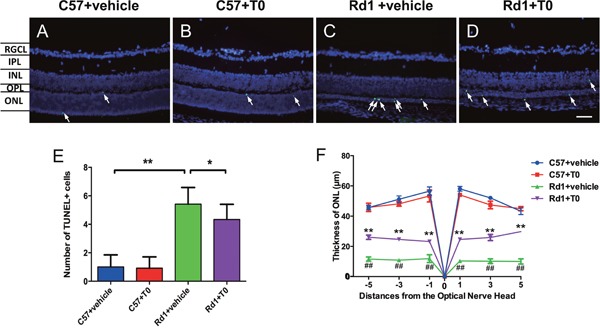
Effects of T0 on apoptosis and thickness of the C57/rd1 retina outer nuclear layer (ONL) at P14 The TUNEL assay was performed at P14. **(A-D)** TUNEL-positive cells (green) are sparsely dispersed in the ONL of C57 mice but densely dispersed in the ONL of rd1 mice. DAPI staining (blue) was shown as a guide for retina layers. **(E)** There is no significant difference in the TUNEL-positive cells in the ONL of C57 mice treated with T0 or vehicle. However, the number of apoptotic cells is remarkably higher in the ONL of rd1 mice than in the age-matched C57 mice, which is significantly reduced by T0 treatment. **(F)** There is no significant difference in the thickness of the ONL of C57 mice treated with vehicle or T0, and the decrease in the thickness of the ONL of rd1 mice treated with T0 is markedly attenuated compared to that in the vehicle-treated control rd1 mice. RGCL, retinal ganglion cell layer; IPL, inner plexiform layer; INL, inner nuclear layer; OPL, outer plexiform layer; ONL, outer nuclear layer. n=4 in each group. **P<0.01, *P<0.05. Scale bar=50 μm.

The rd1 mouse model is characterized by rapid retinal degeneration and photoreceptor apoptosis in the outer nuclear layer (ONL) beginning on P8 and usually peaking on P14 [2, 23]. Apoptosis of cells in the retina of P14 C57 and rd1 mice were tested with TUNEL staining. There are sparsely scattered TUNEL-positive cells distributed in the ONL of C57 mice treated with vehicle or T0 (Figure 2A, 2B). There is no significant difference in the number of TUNEL-positive cells in the ONL of C57 mice treated with vehicle or T0 (Figure 2E). As a result, there is no significant difference in the thickness of the ONL of C57 mice treated with vehicle or T0 (Figure 2F). The number of TUNEL-positive cells in the ONL of rd1 mice at P14 treated with vehicle is markedly higher than in age-matched C57 mice treated with vehicle (Figure 2C, 2E). As a result, the thickness of the ONL of rd1 mice treated with vehicle is significantly thinner than that of age-matched C57 mice treated with vehicle (Figure 2F). However, 7 successive days of treatment with T0 beginning on P7 markedly reduces the number of TUNEL-positive cells in the ONL of rd1 mice (Figure 2D, 2E), and the decrease in the thickness of the ONL is also markedly attenuated (Figure 2F).

### T0 reduces gliosis in the rd1 mouse model

**Figure 3:**
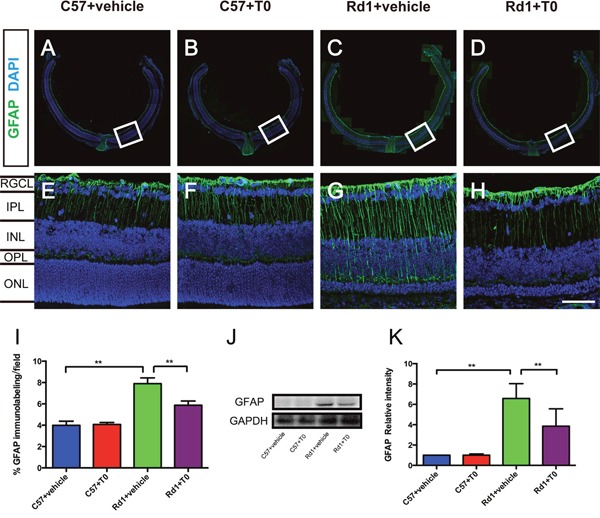
T0 treatment decreases the overexpression of GFAP in rd1 mice **(A-H)** Representative images of GFAP expression in C57 and rd1 mice pretreated with T0 or vehicle. **(E-H)** Images are higher-power views of the boxed areas in A-D. **(I)** Quantitative analysis of the density of GFAP expression in the higher-power fields of each group in E-H. **(J)** Representative western blot of retina lysates from C57 and rd1 mice pretreated with T0 or vehicle. **(K)** The retina from rd1 mice shows significantly more GFAP/GAPDH than that from C57 mice, while T0 treatment significantly reduces this level. RGCL, retinal ganglion cell layer; IPL, inner plexiform layer; INL, inner nuclear layer; OPL, outer plexiform layer; ONL, outer nuclear layer. Data are presented as the mean ± SEM. n=5 mice per group. **P< 0.01. Scale bar in H=75 μm and applies to **(E-H)**.

It has previously been shown that glial fibrillary acidic protein (GFAP) expression in the retina is markedly increased, and the process of gliosis initiated at an early stage in rd1 mice, as a result of retinal injuries and degeneration [13]. In our present study of rd1 mice, immunofluorescence staining shows that GFAP was expressed in the retina of both C57 mice and rd1 mice (Figure 3A-3H), and the percentage of the immunofluorescence intensity per field in the comparable fields of the C57 and rd1 mice treated with vehicle or T0 was analyzed. The results show that the GFAP immunofluorescence intensity is significantly higher in the rd1 mice treated with vehicle than in the C57 mice treated with vehicle (Figure 3G, 3I). Treatment with T0 significantly reduces the GFAP immunofluorescence intensity in the rd1 mice compared with that in the vehicle-treated rd1 mice (Figure 3H, 3I). Western blots confirm the inhibitory effect of T0 on the GFAP level in the retina of rd1 mice (Figure 3J, 3K).

### T0 suppresses the activation of microglia in the retina of rd1 mice

**Figure 4:**
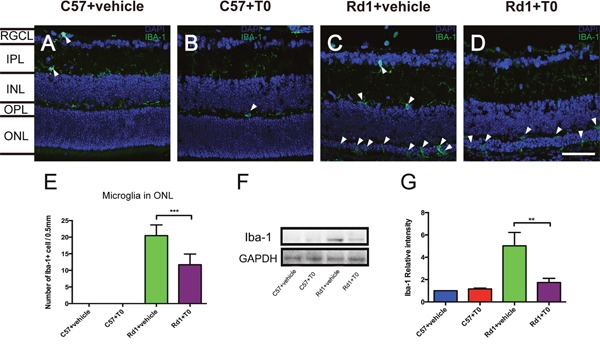
Effect of T0 on Iba-1-positive microglia cells in the retina of C57 mice and rd1 mice at P14 **(A-D)** Representative images of Iba-1-positive cells in the retina of C57 and rd1 mice pretreated with T0 or vehicle. **(E)** Quantitative analysis of the number of Iba-1-positive cells in the ONL of each group. **(F)** Representative western blot of retina lysates from C57 and rd1 mice pretreated with T0 or vehicle. **(G)** The retina from rd1 mice shows significantly more IBA-1/GAPDH than that from C57 mice, while T0 treatment significantly reduces this level. RGCL, retinal ganglion cell layer; IPL, inner plexiform layer; INL, inner nuclear layer; OPL, outer plexiform layer; ONL, outer nuclear layer. Data are presented as the mean ± SEM. n=5 mice per group. ***P< 0.001, **P< 0.01. Scale bar in D=75 μm and applies to **(A-D)**.

Microglia were detected with Iba-1 immunofluorescence staining. Iba-1-positive microglia are mainly distributed in the inner nuclear layers (INL) of P14 C57 mice treated with vehicle (Figure 4A), and the morphology of the microglia remains ramified and slender. Hardly any Iba-1-positive microglia are observed in the ONL of C57 mice on P14 (Figure 4A). Treatment with T0 does not influence the number, distribution or morphology of microglia in the retina of C57 mice on P14 (Figure 4B, 4E).

The number of Iba-1-positive microglia increases significantly in the retina, especially in the ONL of the P14 rd1 mice treated with vehicle, and the morphology of the microglia is changed to the activated amoeboid shape with few processes (Figure 4C, 4E). Treatment with T0 markedly decreases the number of microglia in the ONL of the retina of rd1 mice on P14 (Figure 4D, 4E). Western blot of Iba-1 confirms the trends of the number of microglia in the retina of C57 mice and rd1 mice treated with T0 (Figure 4F, 4G).

### T0 improves the visual function of rd1 mice

**Figure 5:**
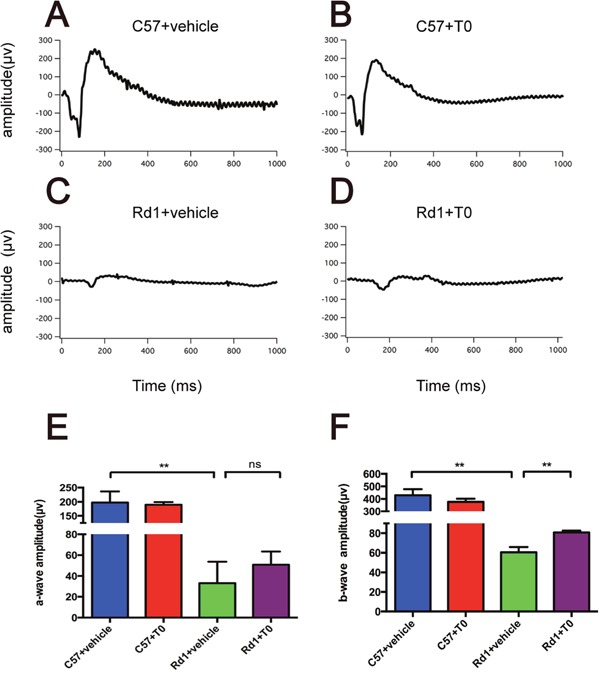
Electroretinogram test of the C57 and rd1 mice at P14 treated with T0 **(A-D)** Representative raw ERG traces recorded from T0- or vehicle-treated C57/rd1 mice showing the definitions for the a- and b-waves. **(E, F)** Significant reduction of a- and b-wave amplitude of rd1 compared with C57 mice treated with vehicle. With T0 treatment, the b-wave of rd1 mice is significantly higher than that of control rd1 mice, while there is no difference in a–wave amplitude between them. n=5 mice per group. Data are presented as the mean ± SEM. n=5 mice per group **P<0.01.

To confirm the protective effect of T0 on the degenerative retina of rd1 mice, scotopic ERGs were tested on P14, just after one week of treatment with T0. As the degeneration of rods progressed, the a-wave amplitude and b-wave amplitude in rd1 mice are dramatically decreased compared with that in the age-matched wild-type mice (Figure 5A-5F). However, T0 treatment rescues the decreased b-wave amplitude but does not increase the a-wave amplitude in rd1 mice compared with their controls (Figure 5C-5F). The administration of T0 does not influence the a-wave amplitude or b-wave amplitude in the wild-type mice (Figure 5E, 5F).

### T0 regulates the inflammatory gene expression and innate immunity in the retina of rd1 mice

**Figure 6:**
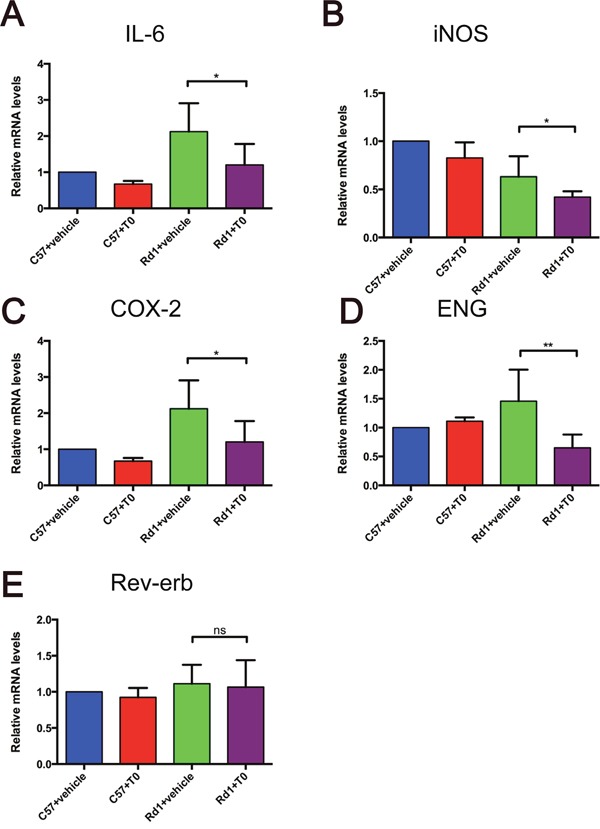
Expression of immune inflammation-related genes in the C57 and rd1 mice at P14 treated with T0 **(A-D)** T0 treatment significantly reduces the expression of mRNA of IL-6, iNOS, COX-2 and ENG in rd1 mice compared with vehicle-treated mice. **(E)** There is no significant difference between the expression levels of Rev-erb in T0-treated and vehicle-treated rd1 mice. Data are presented as the mean ± SEM. n=5 mice per group. *P<0.05, **P<0.01, ns P>0.05.

Previous studies have demonstrated that LXRs are important regulators of inflammatory gene expression and innate immunity [14]. The activation of LXRs can repress the expression of mediators, such as inducible nitric oxide synthase (iNOS), interleukin (IL)-6, and cyclooxygenase-2 (COX-2) [14]. Nuclear orphan receptor Rev-erb can regulate the inflammatory response of murine macrophages through the suppression of IL-6 expression [24]. ENG (Endoglin/CD105), as a part of the transforming growth factor (TGF) receptor complex that binds several members of the TGF-β superfamily, is a direct target of LXRα [25]. To investigate the effect of the activation of LXRs by T0 on the retina of rd1 mice, RT-qPCR of multiple genes were performed. IL-6, iNOS and COX-2 expression are significantly lower in rd1 mice treated with T0 than in vehicle-treated mice (Figure 6A-6C). T0 treatment also decreases the expression of ENG but not Rev-erb in rd1 mice compared those in vehicle-treated rd1 mice (Figure 6D, 6E). The administration of T0 does not influence these genes expression in the wild-type mice (Figure 6A-6E).

### Microarray analysis of T0- or vehicle-treated rd1 mice

**Figure 7:**
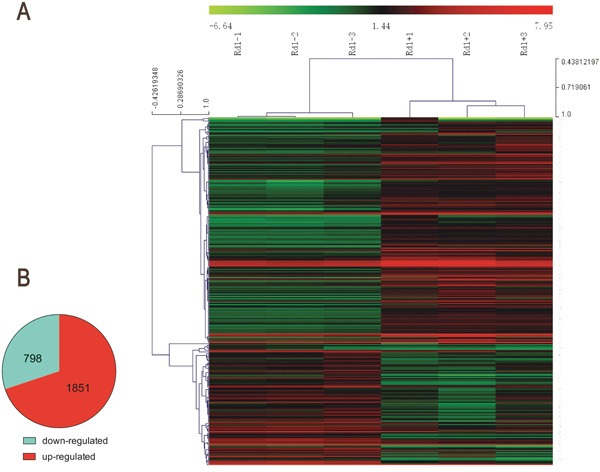
Microarray analysis of T0 affected gene expression in rd1 mice retina at P14 **(A)** Heat map of the top 296 differentially expressed genes (intensity D-value>2100) in 6 rd1 mice exposed to T0 or vehicle as determined by microarray analysis. Red: up-regulation; green: down-regulation. **(B)** Total differentially expressed genes (P≤0.05, absolute fold change≥|±1|).

**Figure 8:**
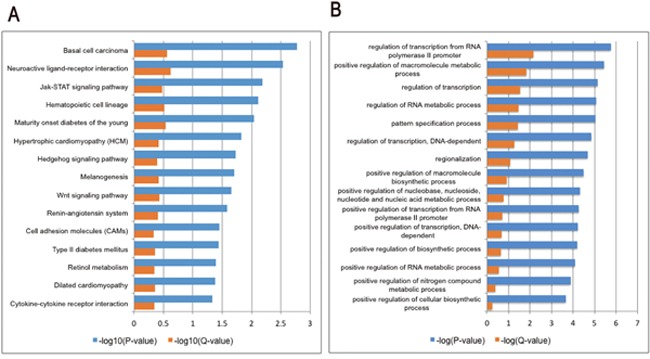
Column charts of the GO and pathway enrichment analyses of DEGs in the retina of T0 treated rd1 mice at P14 **(A)** The most significant 15 pathways from the Kyoto Encyclopedia of Genes and Genomes (KEGG) pathway enrichment analyses. **(B)** The most significant 15 biological processes from the gene ontology (GO) enrichment analyses. P-value and Q-value (Benjamini) represent significance of enrichment.

**Figure 9:**
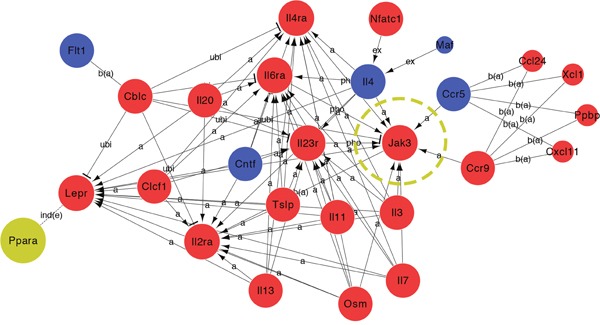
Global Signal Transduction Network analyses of DEGs in the JAK-STAT pathway in the retina of T0 treated rd1 mice at P14 27 DEGs were analyzed by global signal transduction network. Expression levels were shown in blue (down-regulated) and red (up-regulated) while PPAR gene expression didn't get significantly changed. JAK3 plays an important role and carried a strong capability of mediating in the activated pathway.

**Figure 10:**
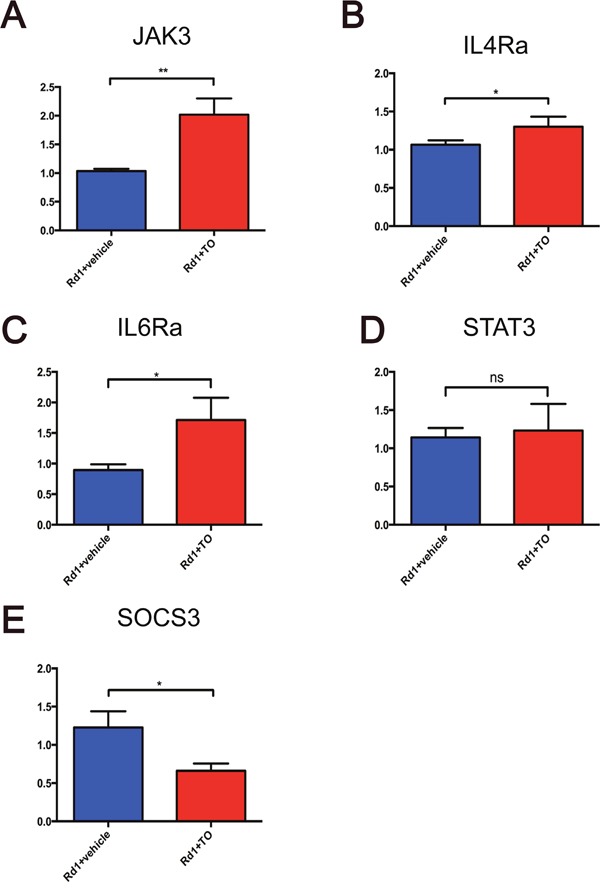
Expression of JAK-STAT pathway related genes in the retina of T0 treated rd1 mice at P14 **(A-C)** T0 treatment significantly reduces the expression of mRNA of JAK3, IL4Ra, and IL6Ra in rd1 mice compared with vehicle-treated mice. **(D)** There is no significant difference between the expression levels of STAT3 in T0-treated and vehicle-treated rd1 mice. **(E)** T0 treatment decreases the SOCS3 expression in rd1 mice retina compared with vehicle-treated mice. Data are presented as the mean ± SEM. n=4 mice per group. *P<0.05, **P<0.01, ns P>0.05.

To gain further insight into the mechanisms by which T0 influences the retina of rd1 mice, RNA was extracted for microarray analysis at P14. As represented in the hierarchical clustering of differentially expressed genes (DEGs) (Figure 7), a set of 2649 DEGs is identified in retinas of rd1 mice, including 1851 up-regulated and 798 down-regulated genes, compared with that of controls. The Kyoto Encyclopedia of Genes and Genomes pathway (KEGG) enrichment and gene ontology (GO) analyses were performed with the DEGs (Figure 8). The three most significantly altered pathways highlighted by KEGG analysis are basal cell carcinoma, neuroactive ligand-receptor interaction, and JAK-STAT signaling pathways (Figure 8A). Promotion of transcription from RNA polymerase II, positive regulation of the macromolecule metabolic process and regulation of transcription are the top 3 hits from the GO biological process analysis (Figure 8B). To locate the key gene of the JAK-STAT pathway, a network analysis was performed (Figure 9). The results show that JAK3 is the signal transmission mediation center in the JAK-STAT pathway of the DEGs. RT-qPCR of multiple genes was performed to confirm the result of microarray and network analysis (Supplementary Table 1). The expression level of JAK3, IL4Ra, IL6Ra (Figure 10A-10C) is significantly increased after the treatment with T0 in rd1 mice retina, the expression of STAT3 (Figure 10D) remains unchanged between two groups. SOCS1 (data not show) is rarely expressed in the retina of rd1 mice while SOCS3 (Figure 10E) expression is markedly decreased by the treatment of T0.

## DISCUSSION

In the present study, we found that activation of LXR with the synthetic agonist T0 significantly reduced the apoptosis of photoreceptors in the ONL of rd1 mice, thus delaying the degeneration of the retina. At the same time, T0 was observed to suppress gliosis in the degenerative retina of mice, alleviate the activation of microglia and decrease the level of pro-inflammatory factors. These results suggested that the protection effect of T0 in rd1 mice was through the immunological modulation of LXRs. The JAK-STAT pathway was involved in the effect of T0 on the degenerative retina.

The synthetic non-subtype selective agonist T0901317 activates both LXRα and LXRβ. As a result, the expression of the target genes of LXRs ABCA1 and ABCG1 are distinctly increased. Usually, the agonists enhance the expression of LXRs after they bind to their receptors, and it showed in present study that the expression of LXRβ was markedly increased when the mice received the administration of T0, however, the expression of LXRα in the retina remained unchanged. LXRα is considered to play a key role in cholesterol homeostasis, while LXRβ is reported to modulate the function of immune system and CNS. LXRβ is relatively highly expressed than LXRα in CNS [14]. We speculated that the unchanging of LXRα expression might be a result of competitive inhibition of binding to the agonist between LXRα and LXRβ in the retina. In the study of Zheng et al, when the T0 was used to protect the inner retinal damage induced by N-methyl-D-aspartate, they also found that the expression of LXRα was not increased while the LXRβ expression was markedly increased [20]. In future, as the development of a more specific and potent subtype selective LXR agonist, the underlying mechanisms of LXRs and RD may be clarified.

As one of the most studied retinal degeneration models, the rd1 mouse shows significant rod photoreceptor apoptosis as early as P10 [2, 26] that peaks at P12–14 [1] and is completed by P21 [27]. To observe the protective effect of an LXR agonist on rod photoreceptors of rd1 mice, we injected T0 on P7, just before the initiation of rod photoreceptor apoptosis. On P14, after 7 days of T0 treatment, the TUNEL-positive apoptosis cell number in the ONL was markedly lower than that of the vehicle-treated rd1 mice. As a result, the thickness of the ONL in the center and peripheral retina of rd1 mice was markedly larger in the T0 treatment group than that in the vehicle-treated group, suggesting that activation of LXRs protected the photoreceptor cells from apoptosis. A similar effect of T0 is also observed in an acute model of retinal ganglion cell (RGC) death induced by N-methyl-D-aspartic acid (NMDA), in which intravitreal injected T0 significantly rescues NMDA-induced cell loss as shown with Hematoxylin and eosin staining, TUNEL staining and NeuN staining [20]. Although the pathological mechanisms of RP and NMDA-induced neurotoxicity are substantially different, activation of LXRs seems to produce a similar protective effect on the neural cells in the retina.

The apoptosis of photoreceptors in the rd1 mouse caused by the accumulation of cGMP results from the impairment of Pde6β [26]. Treatment with LXR agonists, including T0 and GW3965, increases the expression of genes coding for activators of photoreceptor guanylate cyclase and the Pde6h subunit in the neural retina of the zebrafish [17], implying that LXR agonists temporarily clear the accumulated cGMP in retina of rd1 mice. However, LXR agonist treatment does not fundamentally alter the apoptotic fate of photoreceptors since the mutation of Pde6β has not been corrected in the rd1 mice.

In retinal degeneration, gliosis of Müller cells down-regulates their cytoprotective function and contributes to edema development, neuronal hyperexcitation and glutamate toxicity, which is a major cause of neuronal degeneration [28]. Increased immunoreactivity for GFAP is a well-known marker for gliosis, especially in Müller cells [29]. Our research indicated that T0 treatment significantly reduced the expression of GFAP in the retina of rd1 mice, which suggested that T0 alleviated gliosis of Müller cells, thus resulting in protection of the retina. Additionally, glial scars established by Müller cell gliosis could inhibit regular tissue repair and the regeneration of the retina [30]. T0 may become a new drug for improving the microenvironment in retinal degeneration.

The resident microglia in the retina play a surveillance role in maintaining the homeostasis of the immune microenvironment through the presentation of antigens, phagocytosis of debris, and the release of neuroprotective factors and anti-inflammatory factors [31]. The apoptosis of photoreceptors during the development of RD was reported to trigger the activation of resident microglia [7, 11], and our previous results confirmed this [2]. In the present study, we observed that T0 treatment suppressed the activation of microglia in the degenerative retina, which might prevent the degenerated or normal photoreceptors from being phagocytosed. Since previous studies have demonstrated that activation of LXRs is effective in advanced basal cell carcinoma [32], which may not be involved in retinal degeneration of rd1 mice.

The mitogen-activated protein kinases (MAPKs) and nuclear factor-kappa B (NF-κB) are regarded as the two classical inflammatory pathways that are involved in the neuroimmunomodulation of glia cells in the CNS. Besides, recently Kim et al shows that JAK-STAT, a new inflammatory pathway, is vital in activating inflammatory response of microglia in the brain. JAK-STAT pathway can be activated by LPS, IFNγ, which results in the phosphorylation of STAT molecules, dimers formation and inflammatory genes expression [47]. The JAK-STAT pathway has been demonstrated to mediate the protection of photoreceptors in rd1 mice [33] or light-induced retinal degeneration [34] and be involved in the GFAP expression of Müller cells [35] in the mouse model. The JAK-STAT pathway can mediate the inhibition of inflammation during mouse uveitis [36, 37] and is involved in the inflammatory response in the human AMD eye [38], suggesting that the JAK-STAT pathway plays an important role in visual inflammation. Additionally, STAT signaling plays a central role during retinal degeneration in the rd10 mouse model [39]. A strong induction of JAK3 mRNA levels suggested that JAK3 plays a prominent role in rd1 mouse retinal degeneration [40]. In present study, we demonstrated that JAK-STAT signaling pathway is among the three most significantly altered pathways in the retina of rd1 mice, which were treated with T0. It suggests that the JAK-STAT also plays a key role in the development of RD and it could be modulated by other nuclear receptors (NRs) besides glucocorticoid receptors (GRs).

Recently LXRs are also reported to produce anti-inflammatory effects through transrepression of target genes. It demonstrated that the SUMOylation of LXRs resulted in the tethering of STAT1 to LXRs [45, 46]. By activating with the natural and synthetic LXR agonists such as T0901317 and GW3965, the LXRs are widely used in the experimental treatment of a serial of neurodegenerative diseases including Alzheimer disease, Parkinson disease, Multiple Sclerosis and Huntington's Disease [14]. Our present study has shown that JAK-STAT signaling pathway plays an important role in the protection of the retina of rd1 mice. JAK3, which is reported to be primarily expressed in immune-cells, is prominently increased at P14 (5-fold) of rd1 mice [40]; it suggests that JAK3 is involved in the development of retina degeneration. The microarray data showed that the expression of JAK3 in the retina of rd1 mice was markedly increased after T0 treatment. According to the possible mechanism of LXRs modulating the JAK-STAT pathway [46], we hypothesized that the increase of JAK3 expression was a result of feedback inhibition receding. To test this, the expression of JAK3, STAT3 and their downstream genes SOCS1 and SOCS3 were analyzed with RT-qPCR. It showed that the expression of JAK3 in the retina of rd1 mice treated with T0 was significantly increased compared with those treated with vehicle, while the SOCS3 expression was decreased markedly. The expression of STAT3 was unchanged between the two groups. It is consistent with previous report [40, 46].

In conclusion, JAK3-STAT pathway played a crucial role during the retina degeneration process of rd1 mice; the activation of LXRs protected rd1 mouse retina and rescued visual function by suppressing the immune inflammation mediated by JAK3-STAT pathway. LXRs agonist might become new therapeutic agents for RP.

## MATERIALS AND METHODS

### Animals

Rd1 and C57/BL6 mice were purchased from the Third Military Medical University (TMMU) and housed with a 12-h light/dark cycle and ad libitum access to water and food. All experimental procedures were approved by the Animal Care and Use Committee of the TMMU and carried out in accordance with the guidelines for laboratory animal care and use issued by the Chinese Council on Animal Research and the Guidelines of Association for Research in Vision and Ophthalmology.

### Drug treatment

T0901317 (Cayman, Ann Arbor, MI, USA) was dissolved in 100% dimethylsulfoxide (DMSO) at 1 g/ml and was stored in aliquots at -20°C. Before administration to mice, the DMSO was diluted with PBS to a final concentration of 2% [13, 14].

The day of birth was designated as postnatal day 0 (P0). On P7, pups were randomly divided into four groups: T0-treated rd1 group, vehicle-treated rd1 group (2% DMSO in PBS), T0-treated C57 group and vehicle-treated C57 group. An i.p. injection of T0 (50 mg/kg/d) was given for 7 successive days; control mice received the same volume of vehicle (Supplementary Figure 1).

### Histologic processing

Mice were sacrificed at P14. Eyeballs were enucleated and fixed in 4% paraformaldehyde (PFA) at room temperature for 30 min. The anterior segments were then removed microscopically and fixed in 4% PFA for another 1.5 h. Then, the eyes were infiltrated with 30% sucrose overnight at 4°C and embedded at optimal cutting temperature (OCT) (Sakura) for cryosection preparation. Using a freezing microtome (Thermo), 10-μm-thick sections were cut in a sagittal plane as described previously [41]. Serial sections were collected through the pupillary-optic nerve axis.

The immunofluorescence of sections was analyzed as described previously [2]. Sections were incubated in 0.3% Triton X-100 and 3% bovine serum albumin (BSA) at room temperature for 30 min. Subsequently, the sections were incubated with the primary antibody anti-glial fibrillary acidic protein (GFAP) (1:500; Abcam, Cambridge, UK) or anti-Ionized calcium binding adaptor molecule 1 (Iba-1) (1:500; Wako, Japan) in 1% BSA at 4°C overnight. Then, sections were incubated with 488- or cy3-conjugated secondary antibodies (1:200; Jackson ImmunoResearch, West Grove, PA, USA) for 1 h at 37°C. Before examination with a confocal laser scanning microscope (CLSM, Leica Germany), sections were counterstained with 4′, 6-diamidino-2-phenylindole (DAPI) (Sigma-Aldrich, St. Louis, MO, USA). Additionally, the terminal deoxynucleotidyl transferase UTP nick end labeling (TUNEL) assay was performed to detect the ratio of cell apoptosis with the *In Situ* Cell Death Detection Kit (Fluorescein or TMR, Roche Diagnostics, Germany); the procedure was performed according to the manufacturer's instruction.

All staining (immunofluorescence and TUNEL assay) was viewed and photographed with a Leica TCS SP50 confocal microscope (Leica Microsystems). The thickness of the ONL was analyzed from six locations from the optic nerve head in five retinal sections of five rd1 mice using Photoshop CS5 (Adobe Systems).

The number of Iba-1-positive cells or TUNEL-positive cells in the retina was analyzed using stereological principles on serially cut sections of retina. For every retina sample, all Iba-1-positive cells or TUNEL-positive cells were counted in every 20th section, resulting in the analysis of 15–20 sections. The total number of cells was obtained by multiplying the number of cells by the sampling fractions [42]. For each cell slide analysis, at least seven fields amplified by 400 × were chosen to count.

### Western blot analysis

Western blot analysis was performed according to our previous methods [43]. After eyes were enucleated from sacrificed mice, the retinas were isolated from the globe and mixed in ice-cold tissue lysis buffer (1% PSMF + 99% RIPA). The mix was milled using a glass-stirring rod for 5 min to fully lyse the tissue. The lysates were cleared by centrifugation at 2000 g at 4°C for 30 min, and then the protein concentration of the supernatants was determined using a bicinchoninic acid assay (BCA) (Beyotime, China). As previously reported [2], 30 μg of protein sample from each group was separated using a 15% sodium dodecyl sulfate (SDS) polyacrylamide gel and then transferred to polyvinylidene fluoride (PVDF) membranes (Bio-Rad). After blocking with 5% nonfat milk in tris-buffered saline/Tween (TBST), the membranes were incubated with anti-GFAP (1:500; Abcam), anti-Iba-1 (1:500; Wako) and anti-glyceraldehyde-3-phosphate dehydrogenase (GAPDH) (1:1000; CWbio, China) overnight at 4°C. The next day, the membranes were washed with TBST three times and then incubated with peroxidase-conjugated immunoglobulin G (1:2000; Santa Cruz Biotechnology) as secondary antibodies. Finally, the protein bands were scanned with the Odyssey infrared imager system. Staining was then quantified and analyzed by image lab software using GAPDH as an internal control.

### Real-time quantitative polymerase chain reaction (RT-qPCR)

The RT-qPCR experiment was performed as previously described [18]. In brief, total RNA was extracted using TRIZOL (Sigma Aldrich) according to the instructions of the manufacturer. The concentration of the RNA was measured using a spectrophotometric instrument (NanoDrop). Total RNA (approximately 1-2 μg per 20 μl reaction) was reverse transcribed using a PrimeScript RT Reagent Kit (Takara). qPCR was performed with a CFX96 Real-Time PCR system (Bio-Rad) using a SYBR Green qPCR Mix (Takara) according to the manufacturer's instructions. Relative expression levels were normalized to cyclophilin-A and were calculated using the 2^−ΔΔC (t)^ method. All of the primers were purchased from Sangon Biotech (Supplementary Table 1) The PCR cycling was designed as follows: 5 min at 94°C, 35 cycles of 30 s at 94°C, 30 s at 65°C, 30 s at 72°C, 10 min at 72°C and storage at 4°C.

### Electroretinograms (ERG)

Visual electrophysiological tests were performed as described previously [41, 43]. Before the test, mice at P14 were dark-adapted for nearly 12 h. Intramuscular injections of amine (100 mg/kg) and xylazine (12 mg/kg) were performed for anesthesia. Pupils were dilated with one-drop tropicamide and phenylephrine. Gold wire loops were used as recording electrodes for cornea ERG recordings, and 0.9% saline was frequently applied on the cornea to prevent dehydration and allow electrical contact. Two stainless steel needles inserted under the scleral conjunctiva around the equator of the eyes served as the reference electrodes, and another needle placed in the tail served as the ground. Then, the mice were subjected to sequences of scotopic flashes of -20 and 0 db; the flash interval was set at 60-120 s to avoid any adapting effect from the previous flash. Data were acquired by the Reti-scan system (Roland, Germany) and processed by Igor.

### Microarray analysis

According to our previous methods [44], the total retina RNA was extracted using TRIZOL (Sigma Aldrich). The mRNA was amplified using an Amino Allyl MessageAmp II aRNA Amplification Kit (Ambion, AM1753), labeled with NHS-CyDye (Cy5), and then hybridized to the Mouse Whole Genome OneArrayTM Version 2.1 (PhalanxBiotech Group, Taiwan) according to the manufacturer's protocol. Three biological and technical repetitions were performed. The data were analyzed by the Rosetta Resolver® System (Rosetta Biosoftware). Genes with |log2FC (fold change)| >1 and a false discovery rate (FDR) value <0.05 were selected as differently expressed genes (DEGs). Microarray data was submitted to The Gene Functional Classification Tool of Database for Annotation, Visualization and Integrated Discovery (DAVID, https://david.ncifcrf.gov/home.jsp) to perform gene ontology (GO) and Kyoto Encyclopedia of Genes and Genomes (KEGG) pathway enrichment analyses by corrected P-value and Q-value ≤ 0.05 cutoffs.

### Statistical analysis

The data are presented as the mean ± SEM. Data were analyzed using one-way ANOVA followed by Fisher's protected least-significant difference post hoc tests in RT-qPCR, histologic analysis, ERG analysis and Western blot analysis with the SPSS 21.0 software. For all comparisons, the significance level was set at P < 0.05.

## SUPPLEMENTARY MATERIALS FIGURES AND TABLES


