# Supplementary material for: Activation of liver X receptor delayed the retinal degeneration of rd1 mice through modulation of the immunological function of glia
Source: Oncotarget. 2017 Mar 29;8(19):32068–82. doi: 10.18632/oncotarget.16643 (PMC5458269; doi:10.18632/oncotarget.16643)
Supplement: Supplementary file 1 [file oncotarget-08-32068-s001.pdf]

Activation of liver X receptor delayed the retinal degeneration of rd1 mice through modulation of the immunological function of glia

SUPPLEMENTARY FIGURE AND TABLE

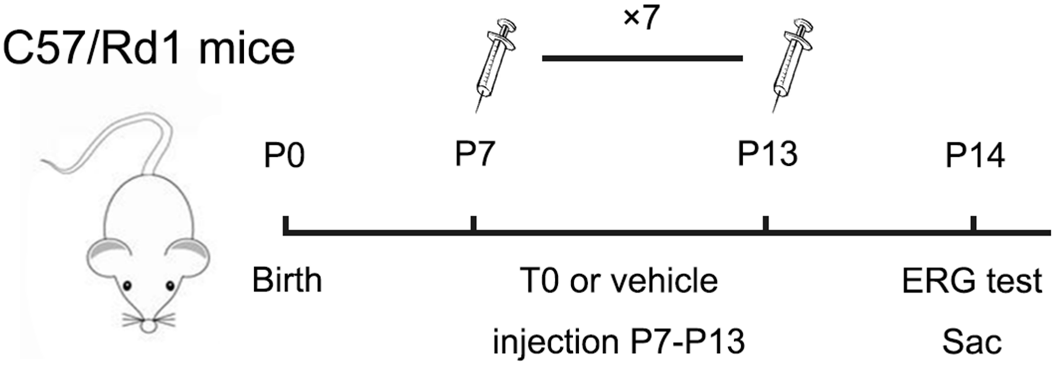

Supplementary Figure 1: Schematic diagram of the experimental procedures. Mice were injected with T0901317 or vehicle for 7 successive days (P7-P13). Animals were sacrificed after the ERG test at P14, and the retinas were removed for immunofluorescence, western blot or RT-qPCR.

Supplementary Table 1: Designing QT-PCR primers

Genes (mouse)	Forward primer	Reverse primer
Cyclophilin-A	CGAGCTCTGAGCACTGGAGA	TGGCGTGTAAAGTCACCACC
LXRα	TCCATCAACCACCCCCACGAC	CAGCCAGAAAACACCCAACCT
LXRβ	TCGCCATCAACATCTTCTCAG	GTGTGGTAGGCTGAGGTGTAA
ABCA1	GGGTGAACGAGTTTCGGTATG	CTGAAGATGCTTGGCTTTGCT
ABCG1	AGAAAGGATGAAGGCAGACGG	TGCTGGGTTGTGGTAGGTAGGG
Rev-erb	GTCCTCGTCTGTTCCATCTTC	AATCTGTGCGGTCACTCTTC
ENG	CACAGTGCTACCATCCCTTAC	GGTCATCCAGTGCTGCTATC
iNOS	CCTCCACCCTACCAAGTAGTAT	GCAAAGGAGGAGAAGGAGAAG
Cox-2	GGCCATGGAGTGGACTTAAA	GATACACCTCTCCACCAATGAC
IL-6	CTTCCATCCAGTTGCCTTCT	CTCCGACTTGTGAAGTGGTATAG
JAK3	CACAGTGCATGGCCTATGAT	AGGTGTGGGGTCTGAGAGG
STAT3	CAAAACCCTCAAGAAGCCAAGG	TCACTCACAATGCTTCTCCGC
SOCS1	TCGACTGCCTTTTCGAGCTG	GGAAGGGGAAGGAACTCAGC
SOCS3	ACATCTATTCTGGGGGCGAG	AACTCCCGAATGGGTCCAGG
TGF-β	CTGTCCAAACTAAGGCTCGC	GTTGTTGCGGTCCACCATTAG
IL4RA	GAAGTGGGCAGGCTTCTGTAT	TAGGCAGGATTGTCTGCAAGG
IL6RA	CACTGAAGCCGACCTTCCTT	TGTGTGCTGATCTCGTGGTG
